# Factors affecting acceptability of an email-based intervention to increase fruit and vegetable consumption

**DOI:** 10.1186/1471-2458-14-1020

**Published:** 2014-09-30

**Authors:** Emily J Kothe, Barbara A Mullan

**Affiliations:** School of Psychology, Deakin University, Melbourne, VIC 3125 Australia; School of Psychology and Speech Pathology, Curtin University, Perth, Australia

## Abstract

**Background:**

Fresh Facts is a 30-day email-delivered intervention designed to increase the fruit and vegetable consumption of Australian young adults. This study investigated the extent to which the program was acceptable to members of the target audience and examined the relationships between participant and intervention characteristics, attrition, effectiveness, and acceptability ratings.

**Methods:**

Young adults were randomised to two levels of message frequency: high-frequency (n = 102), low-frequency (n = 173). Individuals in the high-frequency group received daily emails while individuals in the low-frequency group received an email every 3 days.

**Results:**

Individuals in the high-frequency group were more likely to indicate that they received too many emails than individuals in the low-frequency group. No other differences in acceptability were observed. Baseline beliefs about fruit and vegetables were an important predictor of intervention acceptability. In turn, acceptability was associated with a number of indicators of intervention success, including change in fruit and vegetable consumption.

**Conclusions:**

The findings highlight the importance of considering the relationship between these intervention and participant factors and acceptability in intervention design and evaluation. Results support the ongoing use of email-based interventions to target fruit and vegetable consumption within young adults. However, the relationships between beliefs about fruit and vegetable consumption and acceptability suggest that this intervention may be differentially effective depending on individual’s existing beliefs about fruit and vegetable consumption. As such, there is a pressing need to consider these factors in future research in order to minimize attrition and maximize intervention effectiveness when interventions are implemented outside of a research context.

## Background

In Australia, it is recommended that adults consume at least two servings of fruit and five of vegetables daily [1]. As in most other developed countries [2], consumption is low across the population and young adults have particularly low levels of consumption [2–4]. Many young people cannot correctly report the guidelines for fruit and vegetable consumption and hold misconceptions which are likely to lessen their ability to meet recommendations [5].

Fresh Facts is a 30-day email-based program designed to increase Australian young adults’ fruit and vegetable intake [6–8]. The intervention is based on the theory of planned behaviour TPB; [9, 10] and has previously been shown to be successful in bringing about increases in individual’s attitudes towards eating fruit and vegetables and in increasing the perceived social pressure to consume fruit and vegetables [7].

Pilot testing indicated that an earlier 15-day version of Fresh Facts was acceptable to participants [6]. However, a second phase of acceptability testing was needed in light of significant changes made to the intervention program between the 15-day and 30-day programs (see Table 1) and the need to consider the impact of message frequency on acceptability of the longer more intense intervention [6]. Thus this paper both replicated a previous evaluation of the program and expands on the 30-day version by considering the relationship between participant and intervention characteristics (including intervention intensity), attrition (an indirect measure of feasibility), effectiveness, and acceptability/feasibility ratings.

**Table 1:** **Comparison between Fresh Facts (15 day) and Fresh Facts (30 day)**

	Fresh Facts (15 day)			Fresh Facts (30 day)	
	High Frequency	Medium Frequency	Low Frequency	High Frequency	Low Frequency
Intervention target(s)	Perceived behavioural control			Perceived behavioural control, attitude, subjective norm	
Email frequency	15	10	5	27	9
Total number of intervention messages	15	10	5	27	27
Intervention messages per email	1	1	1	1	3
Email format	Text only	Text only	Text only	HTML and Text	HTML and Text
Images included in emails	No	No	No	Yes	Yes

## Methods

### Participants and procedure

Data were collected from undergraduate students enrolled in an introductory psychology course at an Australian University. Students received course credit for participation in the study. All aspects of the study occurred online and could be completed from any computer with internet access. Written informed consent for participation was obtained from all participants, who were all adults aged 18 years and older.

Participants were randomly assigned to one of three groups: (1) low-frequency intervention (2) high-frequency intervention or (3) control. The acceptability data included in this manuscript was drawn from two studies that sought to assess the efficacy of the Fresh Facts intervention [7, 8]. The results of both studies have been published elsewhere [7, 8]. The second of these studies included a control group (who received no intervention emails). Participants randomised to that group did not complete any measure of intervention acceptability and so were not included in the analyses reported in this manuscript. As such, this study includes individuals randomised to either intervention condition in either of the Fresh Facts efficacy studies. Both studies were approved by the University Human Research Ethics Committee.

Participants completed measures of fruit and vegetable intake (servings/day) and TPB variables (intention, subjective norm, perceived behavioural control and attitude) at baseline and immediately post-intervention.

TPB variables were measured using the same measure described in previous evaluations of the Fresh Facts intervention [8]. Intention, attitude, subjective norm and PBC were all assessed using a 100 point visual analogue scale (where higher scores indicated stronger/more positive levels of that construct). Internal consistency for the TPB measures was good (Cronbach’s alphas = .718 - .929).

As in evaluations of the efficacy of Fresh Facts [8], fruit and vegetable consumption was measured using a brief self-report measure of previous day consumption (e.g. *How many servings of fruit did you eat yesterday?*). Scores were summed to create a composite score of the previous day fruit and vegetable consumption.

Intervention acceptability was assessed using an established measure [6, 11] immediately post-intervention. This measure assessed participants beliefs about the intervention on a 6 point Likert scale (e.g. *I think the email messages were… interesting:* 1 = strong disagree, 6 = strongly agree). Intervention materials were delivered to the participant’s nominated email address on days 1–30.

### Intervention

This study tested a 30-day version of Fresh Facts [7, 8]. Since intervention intensity is a crucial feature of the design and implementation of any intervention which includes repeated contact with participants [12], this study investigated the impact of two levels of intervention intensity on acceptability. Participants in the high-frequency group received 27 intervention emails (each containing one intervention message) over the study period, while participants in the low-frequency group received 9 emails (each containing three intervention messages) over the same time period. Content was matched across groups. Table 2 provides examples of how each of the TPB constructs was targeted within the intervention messages. A sample of messages from the intervention have been published previously [7].

**Table 2:** **Example intervention messages included in Fresh Facts**

TPB variable targeted	Example of how construct was targeted within Fresh Facts^*^	Example intervention text targeting this construct
Attitude		
	Factual information about the link between fruit and vegetable consumption and health outcomes from a number of different sources (e.g. “experts” and same age peers) was provided over the course of the intervention	“In Australia, experts agree that you should enjoy a wide variety of nutritious foods including plenty of vegetables, legumes, and fruit. Australian health professionals recommend eating at least 2 serves of fruit and 5 serves of vegetables”
		“Eating more fruit and vegetables makes it easier to control my weight and keeps my skin clear. Eating plenty of fruit and vegetables help me look and feel my best”
Subjective norm		
	Participants were provided information same age peers approval of fruit and vegetable consumption	“Over 80% of young adults think their peers should eat at least 2 servings for fruit and 5 servings of vegetables every day”
	Stories from other young people were included to provide information about the fruit and vegetable consumption of same age peers	“I eat at least 2 fruit and 5 veg every day – and I think everyone should as well”
	Individuals were prompted to compare their own fruit and vegetable consumption to other people they knew and to seek advice and support from individuals who were consuming high quantities of fruit and vegetables	“If there are people in your life who are especially good at eating well, why not ask them how they do it? Talking to others can give you ideas about how to improve your own habits”
Perceived behavioural control		
	Fresh Facts messages emphasised that consumption of fruit and vegetables was easy to perform and achievable for the individual	“Eating the recommended daily intake of fruit and vegetables is not a difficult task. You can do this very easily… you can do it. So do it this week”
	During Fresh Facts development young adults reported that storage of fresh food was a major barrier fruit and vegetable consumption. Participants were provided with instruction and “tips” designed to encourage consumption of fruit and vegetables in spite of this barrier	“Some people say that fruits and vegetables are hard to store – but storage is a snap if you shop and eat smart. You can make up your daily intake of fruit and vegetables with a variety of fresh, frozen, tinned, and dried foods”

*Note examples are not exhaustive; each construct was targeted in a number of different ways over the course of the intervention.

### Data analysis

Differences in attrition and acceptability between the low- and high-frequency interventions were assessed using independent samples t-tests and chi-squared tests. Chi-squared tests and Pearson’s correlations were used to assess the relationships between acceptability ratings, baseline characteristics and change scores for fruit and vegetable consumption and TPB variables.

## Results

A total of 275 participants were sent intervention emails. Age ranged from 18–25, with a mean age of 18.92 (*SD* = 1.37). The majority (77.3%) were female. Sample characteristics are presented in Table 3. Follow-up measures were completed by 217 participants, an attrition rate of 21%. The intervention was highly acceptable to participants (Table 2), ratings for interesting, credible, logical, easy to understand, personally relevant and useful were over 80%. However, some rated it too long, annoying, and containing too many emails, and 10% of participants rated it as confusing.

**Table 3:** **Demographic characteristics of participants at baseline and attrition by condition**

Demographic Characteristic		Low-Frequency		High-Frequency		Differences between conditions
		**Mean**	**SD**	**Mean**	**SD**	***p***
Age (years)		18.88	1.28	18.99	1.51	.533
Baseline fruit and vegetable intake (servings/day)		4.59	2.10	4.49	2.42	.697
		N	%	N	%	*p*
Gender						.961
	Female	132	77.2	79	77.5	
	Male	39	22.8	23	22.5	
Living Situation						.824
	With parents	131	77.1	78	77.2	
	With friends	13	7.6	9	8.9	
	Residential college	12	7.1	6	6	
	Alone	10	5.9	7	7	
	With partner	4	2.4	1	1	
Ethnicity						.783
	Australian	71	41.8	37	36.6	
	Northeast Asian	49	28.8	37	26.7	
	Southeast Asian	16	9.4	12	11.9	
	Southern and Eastern European	9	5.3	7	6.9	
	Southern and Central Asian	10	5.9	6	5.9	
	Northwest European	6	3.5	4	4	
	North African and Middle Eastern	5	2.9	5	5	
	New Zealander, Maori or Pacific Islander	2	1.2	2	2	
Attrition		37	21.4	21	20.6	.502

Participants were randomised to the two intervention groups as follows: high-frequency (n = 102), low-frequency (n = 173). Differences in attrition and acceptability between the low- and high-frequency interventions were assessed using independent samples t-tests and chi-squared tests. There were no significant differences in attrition between the low- and high-frequency interventions (see Table 3). The only significant difference in acceptability between the two versions was for the “too many emails” rating (see Table 4). Individuals in the high-frequency group were more likely to indicate that they received too many emails than individuals in the low-frequency group.

**Table 4:** **Acceptability ratings by condition**

	Low-Frequency	High-Frequency	Low-Frequency	High-Frequency
	Mean (SD)		% Agreed	
Interesting	4.38 (1.03)	4.27 (1.14)	84.6	80.2
Credible	4.58 (0.90)	4.35 (1.06)	91.2	86.4
Logical	4.85 (0.73)	4.75 (0.81)	97.1	95.1
Easy to understand	5.18 (0.71)	5.23 (0.71)	97.8	100
Personally relevant	4.26 (1.04)	4.19 (1.21)	80.1	81.5
Useful	4.44 (1.07)	4.19 (1.15)	86.0	81.5
Complete	4.18 (1.09)	4.12 (1.02)	75.7	76.5
Too long	2.89 (1.27)	2.67 (1.25)	33.8	23.5
Annoying	3.16 (1.32)	3.35 (1.54)	39.7	48.1
Too many emails^a, b^	3.29 (1.28)	3.96 (1.44)	44.9	69.1
Confusing	2.04 (0.94)	2.21 (1.07)	8.8	12.3

^a^Significant differences in mean ratings between high- and low-frequency interventions.

^b^Significant differences in proportion of sample who agreed with statement between high- and low-frequency interventions.

There were no differences in acceptability ratings on the basis of age, gender, ethnicity or living situation. Nor was there any relationship between fruit and vegetable consumption at baseline and any rating of intervention acceptability (Table 5). Baseline beliefs about fruit and vegetables were an important predictor of intervention acceptability (see Table 5). Intention, attitude, subjective norm, and perceived behavioural control at baseline all predicted at least one rating of intervention acceptability. Acceptability was also associated with a number of indicators of intervention success, specifically change in behaviour, attitude, subjective norm and perceived behavioural control (see Table 6). For example, participants who rated the intervention as more interesting had greater increases in behaviour, attitude, and perceived behavioural control.

**Table 5:** **Pearson Correlations between beliefs about fruit and vegetable consumption at baseline and intervention acceptability ratings**

	Baseline behaviour	Baseline intention	Baseline attitude	Baseline subjective norm	Baseline PBC
Interesting	.024	.191**	-.057	.084	.032
Credible	.099	.235**	.087	.180**	.141*
Logical	.129	.199**	.096	.147*	.167*
Easy to understand	.072	.231**	.115	.131	.230**
Personally relevant	-.102	.088	-.014	.046	-.057
Useful	.01	.190**	.044	.163*	.042
Complete	-.024	.182**	.136*	.142*	.124
Too long	.041	-.109	-.178**	.065	-.117
Annoying	-.002	-.167*	.009	-.063	-.023
Too many emails	.073	-.138*	-.104	-.071	-.076
Confusing	-.066	-.153*	-.330**	-.093	-.193**

*Significant at the p < .05 level; **Significant at the p < .01 level. PBC = perceived behavioural control.

**Table 6:** **Pearson Correlations between change in beliefs about fruit and vegetable and intervention acceptability ratings**

	Behaviour change	Intention change	Attitude change	SN change	PBC change
Interesting	.163*	.045	.263**	.057	.173*
Credible	.002	.017	.112	.07	.056
Logical	-.034	.016	.146*	.073	.032
Easy to understand	.021	-.028	.179**	.131	.034
Personally relevant	.102	.098	.212**	.160*	.175*
Useful	.149*	.058	.212**	.075	.150*
Complete	.146*	.092	.106	.142*	.128
Too long	-.032	-.047	-.192**	-.197**	-.160*
Annoying	-.104	-.013	-.175**	-.119	-.082
Too many emails	-.078	-.009	-.136*	-.056	-.174*
Confusing	.067	.011	-.095	-.134*	-.094

*Significant at the p < .05 level; **Significant at the p < .01 level. PBC = perceived behavioural control.

## Discussion

The aim of the current study was to extend on a previous evaluation of Fresh Facts by evaluating the acceptability of the 30-day version of the program. The relationship between participant and intervention characteristics (including intervention intensity), attrition (an indirect measure of feasibility), effectiveness, and intervention acceptability was also investigated. The findings highlight the importance of considering the relationship between these factors in intervention design and evaluation, and provide valuable information about the acceptability of Fresh Facts.

In particular, when taken in conjunction with the previous evaluation of the acceptability of Fresh Facts [6], the analyses conducted as part of this study support the ongoing use of email-based interventions to target fruit and vegetable consumption within young adults. Both levels of intervention intensity were acceptable to participants within this sample – indicating that young adults were generally receptive to receiving health promotion content using this intervention modality. This is consistent with several recent studies that have shown email-delivered health interventions to be well-received by participants across a range of contexts [13, 14].

Previous researchers have noted a need to investigate the role of message frequency in intervention acceptability [14]. It appears that frequency of the emails in the high-frequency intervention was not acceptable to the majority of subjects in that group. While optimal message frequency is likely to be context specific, the finding that other indicators of intervention acceptability were not influenced by the frequency of intervention contact is pertinent. Given the longer emails included multiple intervention messages within in single email it would be plausible to expect that the combination in emails may influence the perception that those emails were logical, easy to understand, useful, too long, and/or confusing. There was also a risk that a change in frequency could have led to a change in the perception of content quality. However, it does not appear that the increased length, and complexity, of the combined email influenced the perception of the intervention content in this manner. This finding suggests that this intervention modality can be delivered with very frequent contact without compromising the other facets of reported acceptability of the intervention. As such, when seeking to design interventions on the basis of this finding future researchers and practitioners should carefully consider the indictors of intervention acceptability that are most relevant to their intervention aims. However they should consider the evidence that “very frequent” contact is likely to be less acceptable to participants than lower frequency contact.

The relationships between beliefs about fruit and vegetable consumption at baseline and acceptability suggest that this intervention may be differentially effective depending on individual’s existing beliefs about fruit and vegetable consumption. For example, individuals with lower baseline intentions were more likely to find the intervention annoying and confusing, and less likely to find it interesting, logical, easy to understand and useful. The finding that intention, attitude, subjective norm, and perceived behavioural control at baseline were all related to intervention acceptability indicates a potential threat to the dissemination of the intervention, since it would appear that the intervention is best received by those who have the least need for it. Evidence for these kinds of differential effects are not unique to Fresh Facts, indeed evaluation of the X-Pack program, an email-delivered program for smoking cessation targeting college students, found that the intervention was rated as more acceptable by individuals with lower levels of nicotine dependence at baseline [15]. Similarly, the Hello World program – an email-supported health promotion program for Dutch pregnant women was best received by individuals with lower levels of alcohol consumption at baseline [16].

The relationships between baseline beliefs and acceptability ratings are of particular relevance since acceptability ratings were associated with a number of indicators of intervention success. Specifically, the primary intervention outcome (fruit and vegetable consumption) was positively correlated with the extent to which individuals believed the intervention was interesting, useful, and complete and negatively correlated with the view that the intervention too long, annoying, confusing and contained too many emails. Given similar links have been observed in at least one other study [17], these findings suggest a pressing need to consider these factors in order to minimize attrition and maximize intervention effectiveness when interventions are implemented outside of a research context.

### Strengths and limitations of the present study

In addition to the issues discussed above, there are a number of factors that should be taken into account when interpreting these results. Firstly, the results presented here consider the feasibility of the Fresh Facts intervention only in the context of university undergraduate students. While the use of a study population that closely matched to the target population of the intervention is appropriate to the evaluation of the intervention itself – this limits the extent to which relationships reported in this study would be generalizable to other populations. As such, readers should be cautious when seeking to apply the results of this study to other contexts.

This study contributes to a small, but growing, literature on the acceptability of behaviour change interventions. The consideration of the relationship between participant and intervention characteristics, attrition, effectiveness, and intervention acceptability is a relatively new advance within this context and is a strength of this stage of Fresh Facts evaluation. These analyses not only provide valuable information about the acceptability of Fresh Facts but also provide important data about potential threats intervention dissemination that may also be relevant for other intervention programmes.

## Conclusion

Despite the limitations of the current study, these results broadly support the conclusion that this email delivered intervention is an acceptable tool for promoting increased fruit and vegetable consumption. Individuals in the high-frequency group were more likely to indicate that they received too many emails than individuals in the low-frequency group. However it does appear that high frequency messages can be used without adversely influencing non-frequency related indicators of intervention acceptability (such as credibility and personal relevance).
